# TNF-α is responsible for the contribution of stromal cells to osteoclast and odontoclast formation during orthodontic tooth movement

**DOI:** 10.1371/journal.pone.0223989

**Published:** 2019-10-16

**Authors:** Saika Ogawa, Hideki Kitaura, Akiko Kishikawa, Jiawei Qi, Wei-Ren Shen, Fumitoshi Ohori, Takahiro Noguchi, Aseel Marahleh, Yasuhiko Nara, Yumiko Ochi, Itaru Mizoguchi

**Affiliations:** Division of Orthodontics and Dentofacial Orthopedics, Department of Translational Medicine, Tohoku University Graduate School of Dentistry, Sendai, Japan; Charles P. Darby Children's Research Institute, UNITED STATES

## Abstract

Compressive force during orthodontic tooth movement induces osteoclast formation *in vivo*. TNF-α plays an important role in mouse osteoclast formation and bone resorption induced by compressive force during orthodontic tooth movement. Stromal cells, macrophages and T cells take part in TNF-α-induced osteoclast formation *in vitro*. Root resorption caused by odontoclasts is a major clinical problem during orthodontic tooth movement. In this study, we determined the cell type targeted by TNF-α during compressive-force-induced osteoclast and odontoclast formation to elucidate the mechanism of bone and root resorption *in vivo*. An orthodontic tooth movement mouse model was prepared with a nickel-titanium closed coil spring inserted between the maxillary incisors and the first molar. Using TNF receptor 1- and 2-deficient (KO) mice, we found that osteoclast and odontoclast formation was mediated by TNF-α in orthodontic tooth movement. We generated four types of chimeric mice: wild-type (WT) bone marrow cells transplanted into lethally irradiated WT mice (WT>WT), KO bone marrow cells transplanted into lethally irradiated WT mice (KO>WT), WT bone marrow cells transplanted into lethally irradiated KO mice (WT>KO), and KO marrow cells transplanted into lethally irradiated KO mice (KO>KO). Using anti-CD4 and anti-CD8 antibodies, T cells were eliminated from these mice. We subjected these chimeric mice to orthodontic tooth movement. Orthodontic tooth movement was evaluated and tartrate-resistant acid phosphatase-positive cells along the alveolar bone (osteoclasts) and along the tooth root (odontoclasts) were counted after 12 days of tooth movement. The amount of orthodontic tooth movement, and the number of osteoclasts and odontoclasts on the compression side were significantly lower in WT>KO and KO>KO mice than in WT>WT and KO>WT mice. According to these results, we concluded that TNF-α-responsive stromal cells are important for osteoclast and odontoclast formation during orthodontic tooth movement.

## Introduction

Hematopoietic stem-cell-derived osteoclasts play a role in bone resorption and remodeling [1]. Macrophage-colony-stimulating factor (M-CSF) and receptor activator of nuclear factor κB ligand (RANKL) are essential molecules for osteoclast formation [2]. Tumor necrosis factor (TNF)-α can also induce differentiation of osteoclasts [3, 4] and might also induce osteoclasts in erosive bone disease [5].

Orthodontic tooth movement relies on remodeling of the periodontal ligament and alveolar bone using external force. These forces induce neurotransmitters, cytokines, and growth factors, which signal to compressive-force-related osteoclasts and tension-force-related osteoblasts, resulting in bone resorption and formation [6, 7]. Force applied to the compression side of the periodontal membrane induces osteoclasts and the tooth moves as osteoclasts resorb bone.

Root resorption is sometimes observed in the clinic as an undesirable side effect of orthodontic treatment. Although many orthodontists recognize root resorption as a serious problem, it is not yet fully understood. Root resorption is thought to be caused by one or more overlapping factors. Several studies have shown that excessive orthodontic force is an important cause of root resorption [8–10]. Other associated factors [11] include tooth morphology [12], tooth intrusion [13–15], periodontal condition [16], and systemic factors, such as genetics [17], the immune system [18, 19], and bone metabolism [20, 21]. Odontoclasts, the cells that cause root resorption, are thought to be similar to osteoclasts. However, compressive-force-induced odontoclast formation is not fully understood in orthodontic tooth movement.

Orthodontic force induces expression of TNF-α, which plays an important role in orthodontic tooth movement [22–27]. We previously showed that TNF-α plays a significant role in mechanically loaded teeth in an orthodontic tooth movement model in which we used both 55 kDa TNF receptor (TNFR)-1 and 75 kDa TNFR-2 deficient (KO) mice, which showed less tooth movement than wild-type (WT) mice [28, 29].

Cells that are directly involved in osteoclastogenesis include macrophages, stromal cells and T cells, all of which express RANKL [30]. Thus, a deeper understanding of the relationships among these target cells *in vivo* may yield important information for the treatment of bone erosive diseases. The role of these cells in TNF-α-induced osteoclast formation *in vivo* was investigated using bone marrow transplants to determine whether these cells were targets of TNF-α. Hematopoietic cells, including macrophages, were destroyed by a lethal dose of irradiation, but stromal cells survived. Donor bone marrow cells were transplanted into the irradiated recipient mice. Thus, the resulting chimeric mice have stromal cells derived from the recipient and macrophages derived from a donor.

In previous research, using this method with WT and KO mice, four types of chimeric mice were generated as follows: chimeric mice with TNFR-containing macrophages and stromal cells, TNFR-containing stromal cells alone, TNFR-containing macrophages alone, and TNFR-deficient macrophages and stromal cells. T cells were deleted by anti-CD4 and anti-CD8 antibodies after the bone marrow transplantation. TNF-α were injected into the supracalvariae of the chimeric mice and osteoclast formation was observed. The results showed that both macrophages and stromal cells are direct targets of TNF-α, with stromal cells contributing to osteoclast formation more than macrophages[31, 32]. Although the importance of stromal cells and macrophages in TNF-α-induced osteoclast formation has been explained, the contribution of these cells in orthodontic-force-mediated osteoclast formation has not been studied.

Many studies have suggested that T cells regulate osteoclast formation and function [33–35], and that activated CD4^+^ T cells produce osteoclast-related cytokines such as RANKL and IL-17 [36–38]. Th17, which is a T cell that expresses IL-17, enhances osteoclast formation. Although other T-cell-expressed cytokines such as INF-γ, IL-4, IL-10, IL-12 and IL-18 inhibit osteoclast formation [39], it is unclear whether T cells affect orthodontic-force-induced osteoclast formation.

In this study, we used chimeric mice to examine the *in vivo* contribution of each TNF-α target cell type in osteoclast and odontoclast formation during orthodontic tooth movement.

## Materials and methods

### Experimental animals

Male C57BL6/J mice aged 9–10 weeks were obtained from CLEA Japan (Tokyo, Japan) and TNFRs KO mice (*Tnfrsf1a*^*tm1lmx*^*Tnfrsf1b*^*tm1lmx*^) were purchased from the Jackson Laboratory (Bar Harbor, ME, USA). The mice were fed a granular diet (Oriental Yeast, Tokyo, Japan) to prevent them exerting excessive chewing force. The mice were kept in cages in a room maintained at 21–24°C with a 12/12-h light/dark cycle [28, 40, 41]. All experimental procedures conformed to the Regulations for Animal Experiments and Related Activities at Tohoku University, and were reviewed and approved by the Institutional Laboratory Animal Care and Use Committee of Tohoku University, and finally approved by the President of the University.

### Experimental tooth movement

A nickel-titanium (Ni-Ti) closed coil spring (Tomy, Fukushima, Japan) was fixed between the maxillary anterior teeth and the left maxillary first molar to move the molar in a mesial direction under anesthesia. The appliance was fixed with a stainless steel wire (0.01-mm diameter) to a hole drilled through each of the two incisors at the alveolar bone level, and tied to the first molar at the posterior end, as described previously [28, 40, 41] (S1 Fig). According to the manufacturer, the activated appliance exerts a force of approximately 10 g. Four mice were used for each group.

### Bone marrow transplantation

We previously established a bone marrow transplantation method between WT and KO mice [31, 32, 42]. Mice were exposed to 10 Gy total body gamma irradiation, which is a lethal dose. Then, 1 × 10^6^ bone marrow cells from WT or KO mice in 100 μl phosphate buffered saline were intravenously injected via the tail vein in WT or KO mice. To investigate the *in vivo* contribution of TNF-α target cell types to compressive-force-induced osteoclast formation, we generated four kinds of chimeric mice. These were chimeric mice in which WT bone marrow cells were transplanted into irradiated WT mice (WT>WT), WT marrow was transplanted into irradiated KO mice (WT>KO), KO bone marrow cells were transplanted into irradiated WT mice (KO>WT), and KO bone marrow cells were transplanted into irradiated KO mice (KO>KO). To confirm the success of the bone marrow transplantation process, we probed for the presence of TNFRs on osteoclast precursors in the four types of chimeric mice that we generated. WT>WT, WT>KO, KO>WT and KO>KO bone marrow cells were cultured with M-CSF for 3 days. The resultant macrophages were incubated with FITC-conjugated anti-TNFR1 mAb (Abcam, Cambridge, UK) or PE-conjugated anti-TNFR2 mAb (BD Biosciences, San Jose, USA). TNFR expression was determined by fluorescent-activated cell sorting (FACS). Macrophages of WT>WT and WT>KO expressed TNFR1 and TNFR2, but KO>WT and KO>KO did not express TNFR1 and TNFR2 (S2 Fig). These results indicated that the bone marrow transplantation was successful.

### *In vivo* T cell depletion

YTS cells, which secrete anti-CD4 antibodies, and H35 cells, which secrete anti-CD8 antibodies, were kindly provided by Dr. O. Kanagawa (Riken, Tokyo, Japan). YTS cells and H35 cells were intraperitoneally injected into a mouse model, which developed ascites containing anti-CD4 and anti-CD8 antibodies that can deplete T cells as previously described [31, 32, 42]. Each mouse was injected with 50 μl of anti-CD4 and anti-CD8 ascites to delete T cells 7 days before and on the day that tooth movement started. The effect of T cell depletion *in vivo* was confirmed by FACS analysis (S3 Fig). Seven days after a single injection of ascites containing anti-CD4 and anti-CD8 antibodies or vehicle, the spleen was removed. Spleen cells were incubated with FITC-conjugated CD4, CD8, and CD3 antibodies or FITC-conjugated isotype antibodies for 1 hour and then analyzed by FACS.

### Measurement of tooth movement

The animals were euthanized by overdose inhalation of 5% isoflurane after 12 days of tooth movement. After dissection of the maxillary region, the distance between the first and second molars was measured. Impressions of the teeth and the maxilla were obtained with the use of individual trays containing hydrophilic vinyl polysiloxane impression material (EXAMIXFINE Injection Type, GC Co., Tokyo, Japan). After an impression was obtained, the samples were fixed in 4% paraformaldehyde. Tooth movement was assessed by measuring the shortest distance (black double arrow) between the distal marginal ridge of the first molar and the mesial marginal ridge of the second molar (dotted line) under a stereoscopic microscope (VH-7000; Keyence, Osaka, Japan) as described previously [28, 40, 41] (S4 Fig). The left side of the maxilla was used for evaluation in each of the groups.

### Sample preparation for histological observation

The fixed teeth and maxillae were decalcified in 14% ethylenediaminetetraacetic acid (EDTA) for 28 days at room temperature, embedded in paraffin, and sectioned in the horizontal plane at 4 μm. We prepared horizontal sections from the distobuccal root at five levels: 100, 140, 180, 220 and 260 μm apical to the first molar bifurcation area. These sections were deparaffinized, stained for tartrate-resistant acid phosphatase (TRAP) activity and counterstained with hematoxylin. Naphthol-ASMX-phosphate (Sigma-Aldrich; St Louis, Missouri, USA), Fast Red Violet LB Salt (Sigma-Aldrich), and 50 mM sodium tartrate were used for TRAP staining. TRAP-positive multinuclear cells, located in lacunae in the resorbed alveolar bone surface, were counted as osteoclasts using light microscopy as described previously [28, 40, 41]. Furthermore, we identified TRAP-positive multinuclear cells located in lacunae in the resorbed root surface as odontoclasts. TRAP-positive cells on the surface of the alveolar bone and on the mesial side of the distobuccal root were counted, and the mean values in five sections were calculated as described previously [41, 43]. Four mice were used for evaluation in each group.

### Measurement of the root resorption area

The mesial side of the distobuccal root was used for evaluation. We quantified the root resorption area in the odontoclast area by calculating the percentage of the surface resorbed on the compression side of the distobuccal root of the first molar after orthodontic tooth movement as described previously [41, 43] (S5 Fig).

### Statistical analysis

All data are presented as the mean ± standard deviation of independent replicates. To test significance, we performed Scheffe’s F test. P values less than 0.05 were considered significant.

## Results

### TNF-α involvement in osteoclast and odontoclast formation, and bone and root resorption resulting from tooth movement independent of T cells

**Fig 1:**
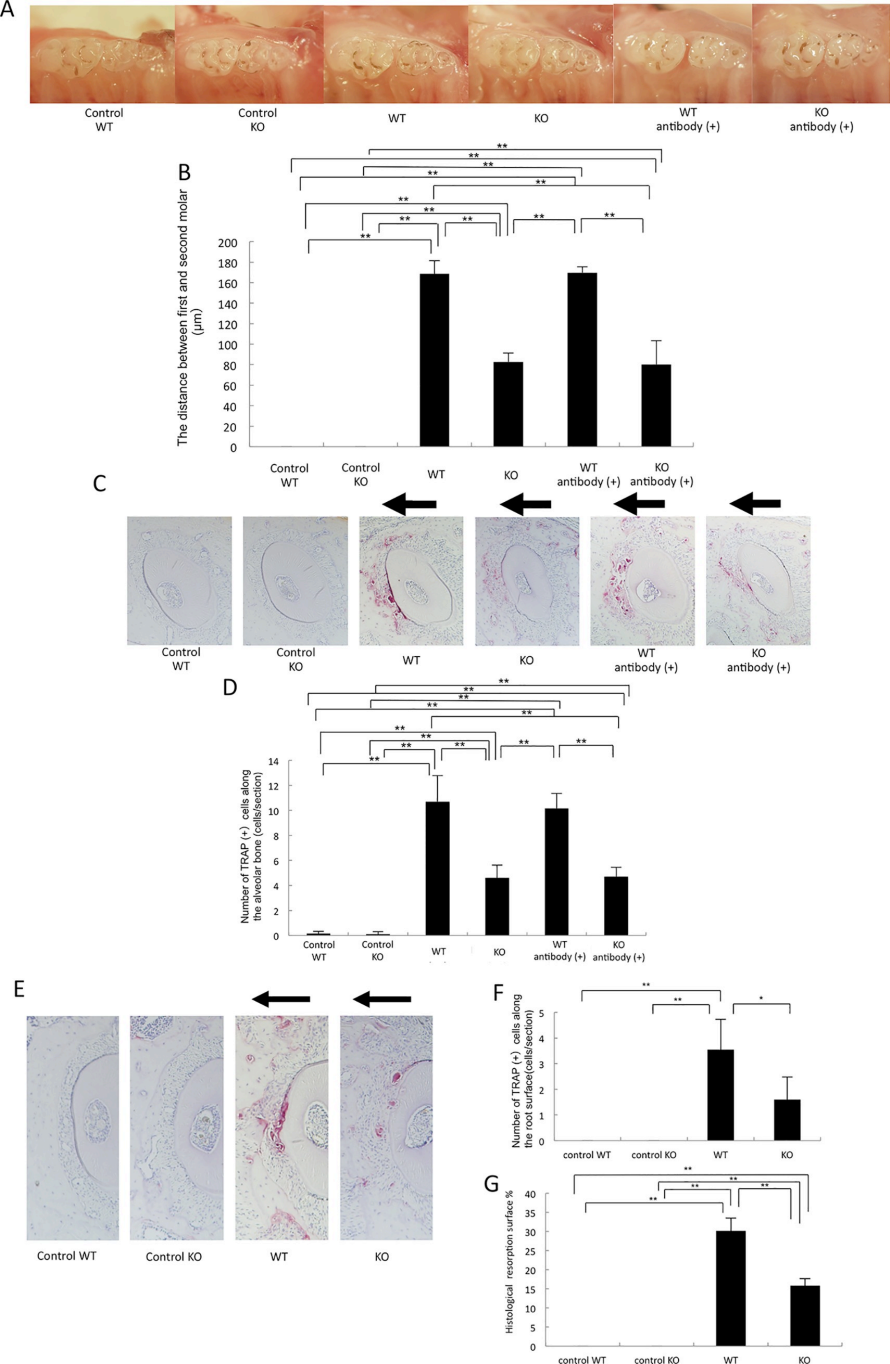
TNF-α involvement with tooth movement, osteoclast and odontoclast formation, and root resorption. (A) Images of teeth after 12 days of experimental loading in WT and KO mice, in unloaded controls of WT and KO mice, and in T cells in depleted and non-depleted WT and KO mice by anti-CD4 and anti-CD8 antibodies. Antibody (+) is T-cell-depleted mice by antibodies. (B) Distance moved by teeth in WT and KO mice and T-cell-depleted WT and KO mice after 12 days of compressive force loading. (C) Horizontal sections of the alveolar bone surface in the left maxillary first molar area. TRAP-stained sections of the distobuccal root of the left maxillary first molar in unloaded controls of WT and KO mice and after 12 days of tooth movement in WT and KO mice and T-cell-depleted WT and KO mice. (D) The number of TRAP-positive osteoclast cells after 12 days of compressive force loading in WT and KO mice and T-cell-depleted WT and KO mice. (E) Horizontal sections of the root surface in the left maxillary first molar area. TRAP-stained sections of the distobuccal root of the left maxillary first molar in unloaded controls of WT and KO mice and after 12 days of tooth movement in WT and KO mice. (F) The number of TRAP-positive odontoclast cells after 12 days of compressive force loading in WT and KO mice. (G) Percentage of root surface resorption on histological sections after 12 days of tooth movement in WT and KO mice. Data are expressed as the mean ± SD (n = 4; **p < 0.01, *p < 0.05).

After 12 days of mechanical loading, the tooth moved 168.8 ± 12.7 μm in WT mice and 82.8 ± 8.6 μm in KO mice (Fig 1A and 1B); therefore, there was less tooth movement in KO mice than in WT mice. After 12 days of mechanical loading, the tooth moved 169.8 ± 5.7 μm in T-cell-depleted WT mice, and 80.3 ± 23.1 μm in T-cell-depleted KO mice. There was no significant difference in the distance of tooth movement between T-cell-depleted and non-depleted WT or KO mice (Fig 1A and 1B). Furthermore, we evaluated the effect of TNF receptor depletion on compressive-force-induced osteoclast formation after 12 days of tooth movement. Paraffin sections of the mesial side of the distobuccal root of the maxillary first molars were examined by TRAP staining. Many TRAP-positive cells were detected on the pressure side of the tooth in the WT mice, while fewer such cells were detected in KO mice (Fig 1C and 1D). Moreover, we investigated the effect of T cells on compressive-force-induced osteoclast formation after 12 days of tooth movement. There was also no significant difference in the number of TRAP-positive cells on the pressure side of the tooth between T-cell-depleted and non-depleted WT mice or KO mice (Fig 1C and 1D). TNF-α affected odontoclast formation during orthodontic tooth movement. After 12 days of experimental tooth movement, the number of TRAP-positive cells along the loaded root surface (odontoclasts) was counted. The number of odontoclasts in KO mice was significantly smaller than that in WT mice (Fig 1E and 1F). We evaluated compression-force-induced root resorption on the mesial side of the distobuccal root under a light microscope. After 12 days of tooth movement, the root resorbed area in KO mice was also significantly smaller than that in WT mice (Fig 1G).

### TNF-α-responsive stromal cells contribute to osteoclast formation and tooth movement *in vivo*

**Fig 2:**
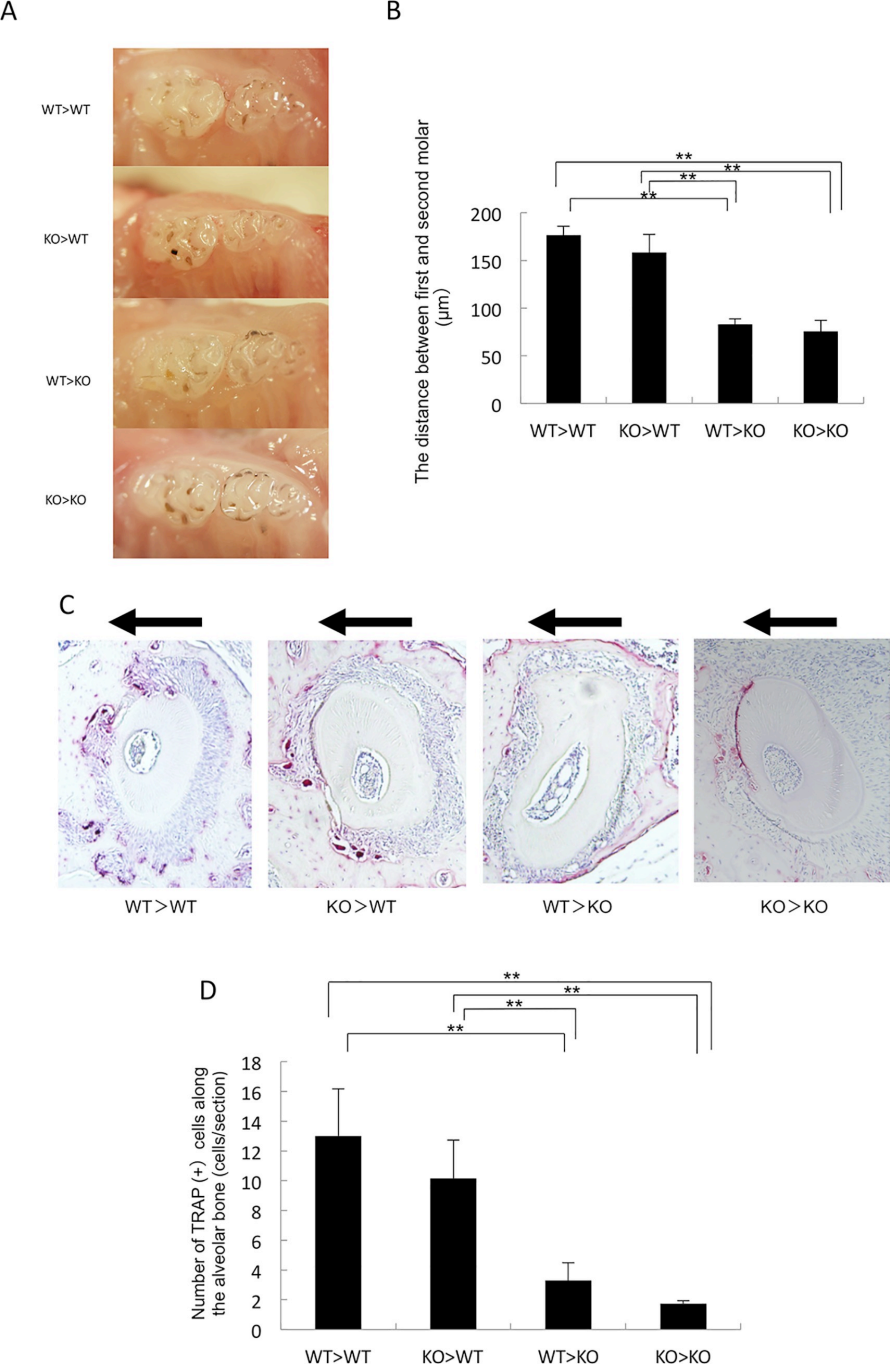
Tooth movement after 12 days of compressive force loading in chimeric WT and KO mice. (A) Images of teeth after 12 days of compressive force loading in chimeric WT and KO mice. (B) Distance moved by teeth after 12 days of compressive force loading in chimeric WT and KO mice. (C) Horizontal sections of the alveolar bone surface of the left maxillary first molar area in chimeric mice after tooth movement. TRAP-stained sections of the alveolar bone surface of the distobuccal root of the left maxillary first molar after 12 days of tooth movement in each chimeric mouse group. (D) The number of TRAP-positive osteoclast cells on the alveolar bone surface of the distobuccal root of the left maxillary first molar after 12 days of tooth movement in each chimeric mouse group. Data are expressed as the mean ± SD (n = 4; **p < 0.01).

TNF-α affects orthodontic tooth movement and mechanically induced osteoclast formation. To investigate the *in vivo* contribution of different cell types, we generated four kinds of chimeric mice. The chimeric mice were: WT>WT, KO>WT, WT>KO and KO>KO. The orthodontic tooth movement was measured and TRAP-positive cells along the compression side of the alveolar bone were counted as osteoclasts after 12 days of tooth movement. The amount of orthodontic tooth movement in WT>KO and KO>KO mice was significantly lower than that in WT>WT and KO>WT mice (Fig 2A and 2B). There was no significant difference between the amount of tooth movement between WT>KO and KO>KO mice or between WT>WT and KO>WT mice. The number of TRAP-positive cells along the compression side of the alveolar bone in WT>KO and KO>KO mice was also significantly lower than that in WT>WT and KO>WT mice (Fig 2C and 2D). There was also no significant difference between the number of TRAP-positive cells along the loaded alveolar bone between WT>KO and KO>KO mice or between WT>WT and KO>WT mice (Fig 2C and 2D).

### TNF-α-responsive stromal cells contribute to odontoclast formation and root resorption during tooth movement *in vivo*

**Fig 3:**
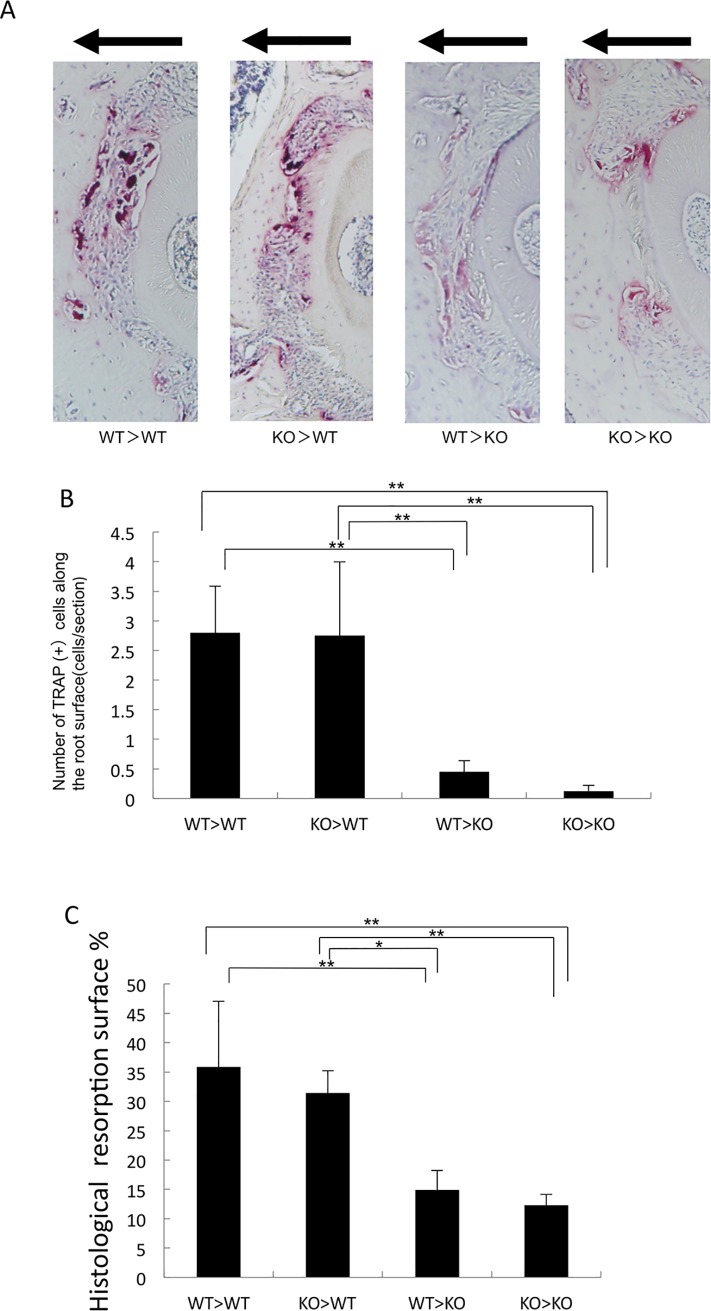
Horizontal sections of the root surface of the left maxillary first molar in chimeric mice after tooth movement. (A) TRAP-stained sections of the surface of the distobuccal root of the left maxillary first molar after 12 days of experimental tooth movement in each chimeric mouse group. (B) The number of TRAP-positive odontoclast cells on the distobuccal root surface of the left maxillary first molar after 12 days of experimental tooth movement in each chimeric mouse group. (C) Percentage of root surface resorption on histological sections after 12 days of tooth movement in each chimeric mouse group. Data are expressed as the mean ± SD (n = 4; **p < 0.01, *p < 0.05).

TNF-α affects odontoclast formation during orthodontic tooth movement. We used four kinds of chimeric mouse described above for evaluation of odontoclast formation and root resorption. After 12 days of experimental tooth movement, TRAP-positive cells along the loaded root surface were counted as odontoclasts. The number of odontoclasts along the loaded root surface in WT>KO and KO>KO mice was significantly lower than that in WT>WT and KO>WT mice (Fig 3A and 3B). There was no significant difference in the number of TRAP-positive cells along the loaded root surface between WT>KO and KO>KO mice or between WT>WT and KO>WT mice (Fig 3A and 3B). We also evaluated the orthodontic-force-induced root resorption on the mesial side of the distobuccal root under a light microscope. After 12 days of tooth movement, the root resorbed area in WT>KO and KO>KO mice was significantly smaller than that in WT>WT and KO>WT mice (Fig 3C). There was also no significant difference in the root resorbed area between WT>KO and KO>KO mice or between WT>WT and KO>WT mice (Fig 3C).

## Discussion

We used an orthodontic tooth movement mouse model to investigate how mechanical force loading induces osteoclast and odontoclast formation. By employing TNFR KO mice, we found that osteoclast and odontoclast formation was mediated by TNF-α during orthodontic tooth movement. Furthermore, we determined the *in vivo* contribution of TNF-α target cell types in osteoclast and odontoclast formation during orthodontic tooth movement using a chimeric mouse model, in which WT or KO marrow was transplanted into lethally irradiated WT and KO mice. We found that TNF-α-responsive stromal cells are important for osteoclast and odontoclast formation during orthodontic tooth movement. To our knowledge, this is the first report of such an analysis using chimeric mice.

We previously showed by immunohistochemistry that TNF-α is expressed in the periodontal membrane during tooth movement [29]. We also used our tooth movement model in KO mice to investigate the role of TNF-α in orthodontic tooth movement [28]. The amount of tooth movement was significantly less in KO mice compared with that in WT mice on days 10 and 12. The total distance moved by teeth and the number of osteoclasts on the compression side were greatest on day 12. Therefore, for this study, we decided that day 12 was the best day for observation. We used KO mice to confirm that TNF-α plays a role in tooth movement. After 12 days of mechanical force loading, the distance moved by teeth and osteoclast formation were less in KO mice than in WT mice. This indicates that TNF-α plays an important role in compressive-force-induced osteoclast formation and bone remodeling during orthodontic tooth movement. Root resorption is sometime observed as an undesirable side effect of orthodontic treatment. In mice, root resorption occurred and odontoclasts were present at the compression side during tooth movement when a 10 *g* force was applied to the tooth by a Ni-Ti closed coil spring [44]. In the present study, a 10 g force was applied to the tooth, and root resorption was observed in many samples. One of the causes of root resorption during orthodontic tooth movement is considered to be too much compressive force [9]. In the present study, our samples exhibited areas of root resorption containing many odontoclasts, indicating that the orthodontic compressive force in this study might be excessive. On day 12, odontoclast formation and the root resorption area was less in KO mice than in WT mice, indicating that TNF-α plays an important role in compressive-force-induced odontoclast formation and root resorption during orthodontic tooth movement.

One of the outcomes of the complex relationship between macrophages and stromal cells is inflammatory osteoclast formation and subsequent bone resorption. TNF-α has been targeted in the treatment of bone-resorbing diseases because of its effect on both cell types [39]. Two methods have been described to explain the role of TNF-α in osteoclast formation; first, by stimulating stromal cells to express RANKL [45], and second, by directly inducing the differentiation of macrophages into osteoclasts. Previous research examined the extent of the contribution of stromal cells versus macrophages in TNF-α-induced osteoclast formation *in vivo* [31, 32]. The studies indicated that stromal cells contribute to TNF-α-induced osteoclast formation in a dose-dependent manner, more than macrophages do, while KO>WT and WT>KO both showed an increase in osteoclast formation compared with KO>KO; however, higher doses of TNF-α were needed to induce osteoclast formation in WT>KO [31, 32]. Therefore, it was concluded that stromal cells response to lower doses of TNF-α while macrophages require higher doses of TNF-α. Similarly, we determined the *in vivo* contribution of each cell type in orthodontic tooth movement, and we used four chimeric mice: WT>WT, WT>KO, KO>WT and KO>KO. The amount of tooth movement and the number of osteoclasts were significantly lower in WT>KO and KO>KO mice than in WT>WT and KO>WT mice. Furthermore, there was no significant difference between WT>KO and KO>KO mice. Only KO>WT chimeric mice, but not WT>KO mice, had a similar increase in the distance of orthodontic tooth movement and the number of osteoclasts generated compared with WT>WT. Furthermore, WT>KO had a significantly shorter distance of orthodontic tooth movement and number of osteoclasts compared with WT>WT. These results indicate that TNF-α expression may be low in orthodontic tooth movement. These findings indicate that TNF-α-responsive stromal cells are important for osteoclast formation during orthodontic tooth movement.

We previously showed that TNF-α is expressed on the compression side of the tooth and plays an important role in tooth movement [28, 29]. Orthodontic tooth movement is often accompanied by root resorption [46]. However, the relationship between odontoclast formation and TNF-α is poorly understood. In this study, odontoclast formation and the area of root resorption in KO mice was less than that in WT mice. These results indicate that TNF-α is concerned with odontoclast formation and root resorption. This is the first report using KO mice and four types of chimeric mice to analyze odontoclast formation and root resorption during tooth movement. The amount of odontoclast formation and root resorption was significantly lower in WT>KO and KO>KO mice than in WT>WT and KO>WT mice. Furthermore, there was no significant difference between WT>KO and KO>KO mice. These results are similar to those for osteoclast formation during tooth movement. These results indicate that TNF-α-responsive stromal cells also play an important role in odontoclast formation and root resorption during orthodontic tooth movement.

## Conclusions

In conclusion, stromal cell TNF-α responsivity is an important factor in osteoclast formation and bone resorption during orthodontic tooth movement. Furthermore, stromal cells can be targeted to prevent odontoclast formation and root resorption.

## Supporting information

S1 Fig Mouse model of orthodontic tooth movement. A nickel-titanium closed coil spring was fixed between the maxillary incisors and the left maxillary first molar with stainless steel wire (0.01 mm diameter) to move the first molar in a mesial direction.
(TIF)

S2 Fig Chimeric mice selectively express TNFRs on osteoclast precursors. WT>WT, KO>WT, WT>KO and KO>KO bone marrow cells were cultured with M-CSF for 3 days. The resultant macrophages were incubated with FITC-conjugated anti-TNFR1 mAb (dashed red lines) or PE-conjugated anti-TNFR2 mAb (dashed red lines). TNFR expression was determined by FACS. TNFR1 solid lines represent cells incubated with FITC-conjugated isotype antibody. TNFR2 solid lines represent cells incubated with PE-conjugated isotype antibody.
(TIF)

S3 Fig T cells are depleted in mice injected with anti-CD4 and anti-CD8 antibodies. Spleen cells were collected 7 days following a single injection of ascites containing anti-CD4 and anti-CD8 antibodies or vehicle. The spleen cells were treated with FITC-conjugated antibodies against CD4, CD8, and CD3 (dashed red lines) or FITC-conjugated isotype antibodies (solid lines) and then analyzed by FACS.
(TIF)

S4 Fig Measurement of tooth movement. Image of the silicone impression (viewed under a stereoscopic microscope) taken after tooth movement for measurement of tooth movement. The amount of tooth movement was measured between the distal marginal ridge of the first molar to the mesial marginal ridge of the second molar at the level connecting the central fossae of the first and second molars (black double arrow).
(TIF)

S5 Fig Evaluation of root resorption on transverse histological sections. The image shows the evaluation of root surface resorption on transverse histological sections. The solid line represents the pressure side of the root surface and the interrupted line is the resorption surface. The root resorption surface was quantified by the percentage of the interrupted line/solid line.
(TIF)
