# Supplementary figures and images for: TNF-α is responsible for the contribution of stromal cells to osteoclast and odontoclast formation during orthodontic tooth movement
Source: PLoS One. 2019 Oct 16;14(10):e0223989. doi: 10.1371/journal.pone.0223989 (PMC6795494; doi:10.1371/journal.pone.0223989)

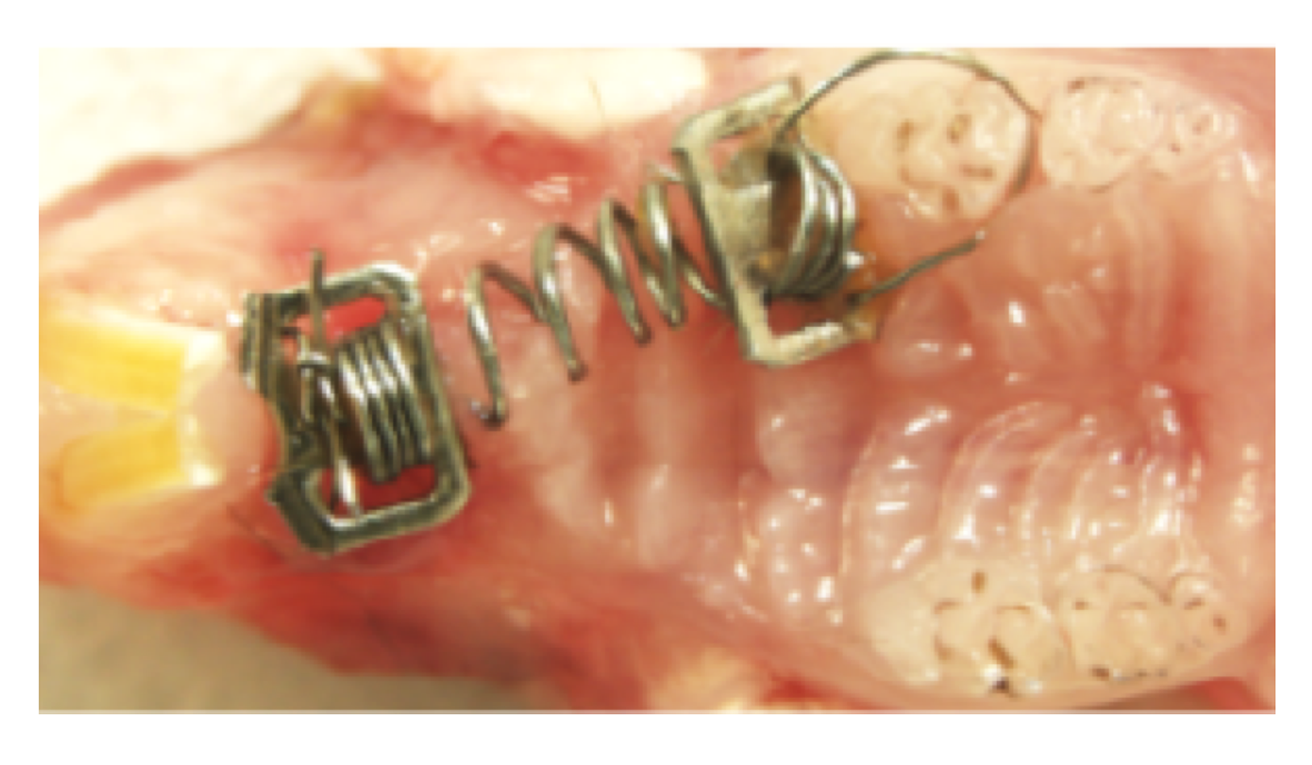

Supplement: S1 Fig — A nickel-titanium closed coil spring was fixed between the maxillary incisors and the left maxillary first molar with stainless steel wire (0.01 mm diameter) to move the first molar in a mesial direction. (TIF) [file pone.0223989.s001.tif]

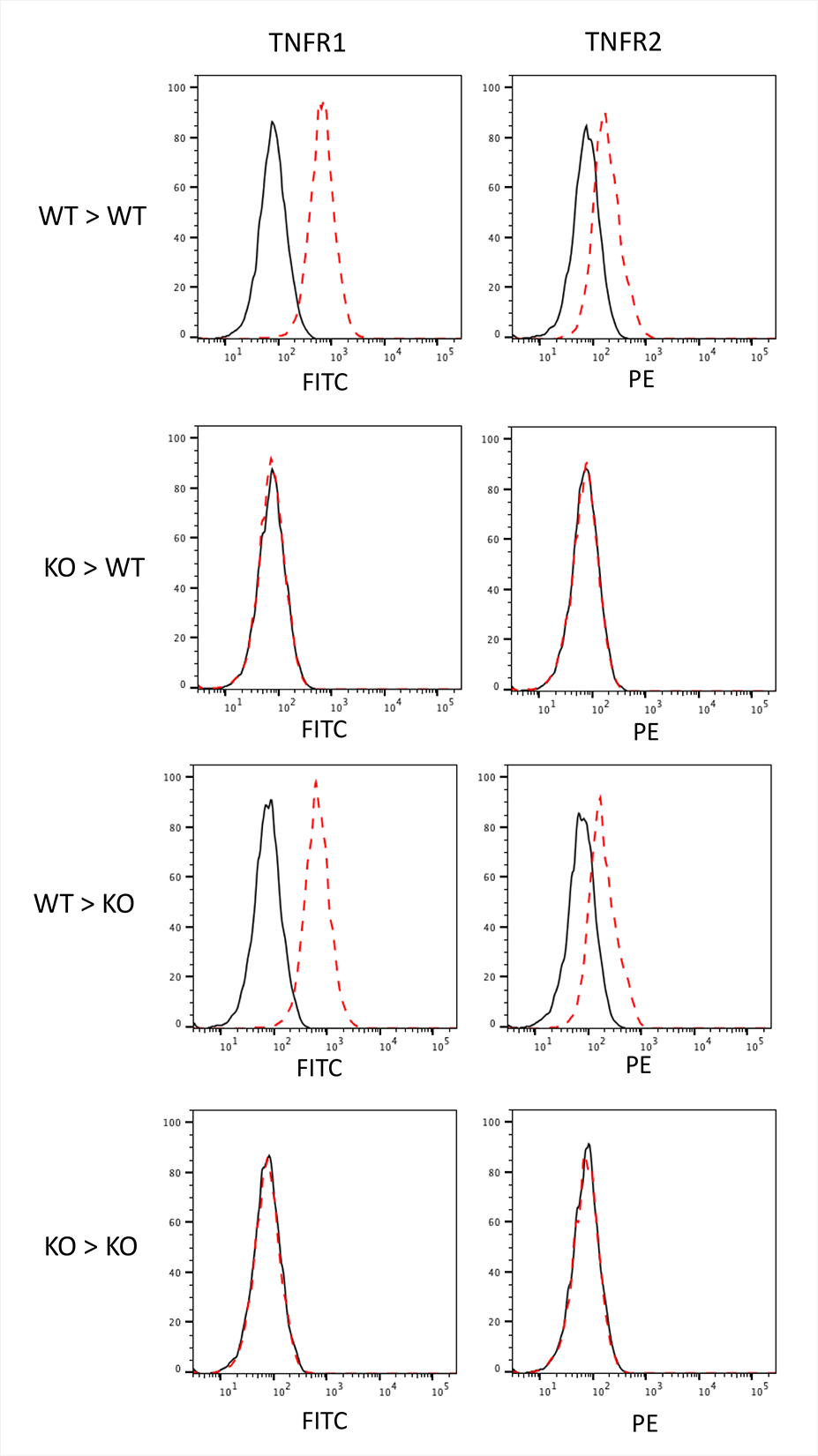

Supplement: S2 Fig — WT>WT, KO>WT, WT>KO and KO>KO bone marrow cells were cultured with M-CSF for 3 days. The resultant macrophages were incubated with FITC-conjugated anti-TNFR1 mAb (dashed red lines) or PE-conjugated anti-TNFR2 mAb (dashed red lines). TNFR expression was determined by FACS. TNFR1 solid lines represent cells incubated with FITC-conjugated isotype antibody. TNFR2 solid lines represent cells incubated with PE-conjugated isotype antibody. (TIF) [file pone.0223989.s002.tif]

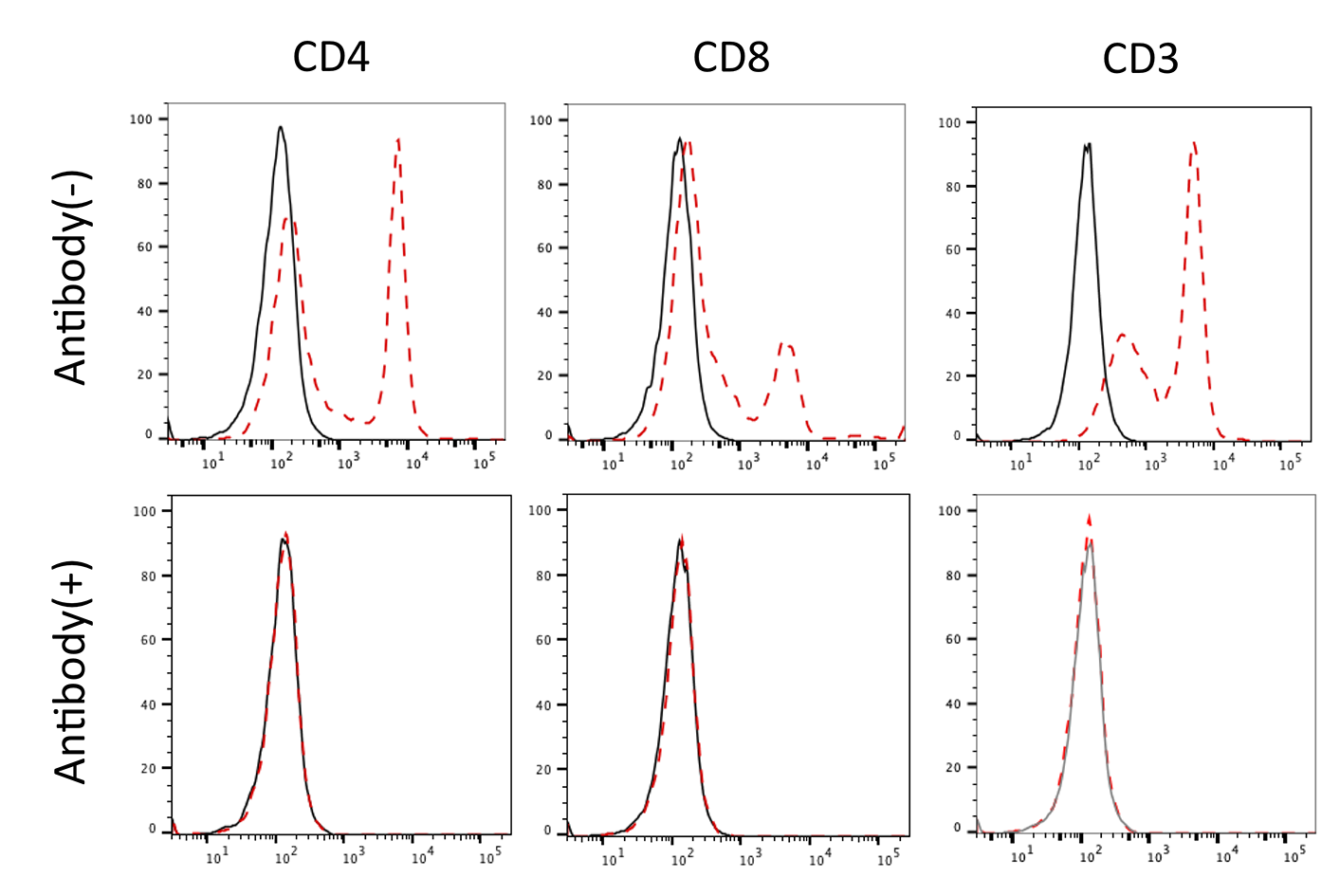

Supplement: S3 Fig — Spleen cells were collected 7 days following a single injection of ascites containing anti-CD4 and anti-CD8 antibodies or vehicle. The spleen cells were treated with FITC-conjugated antibodies against CD4, CD8, and CD3 (dashed red lines) or FITC-conjugated isotype antibodies (solid lines) and then analyzed by FACS. (TIF) [file pone.0223989.s003.tif]

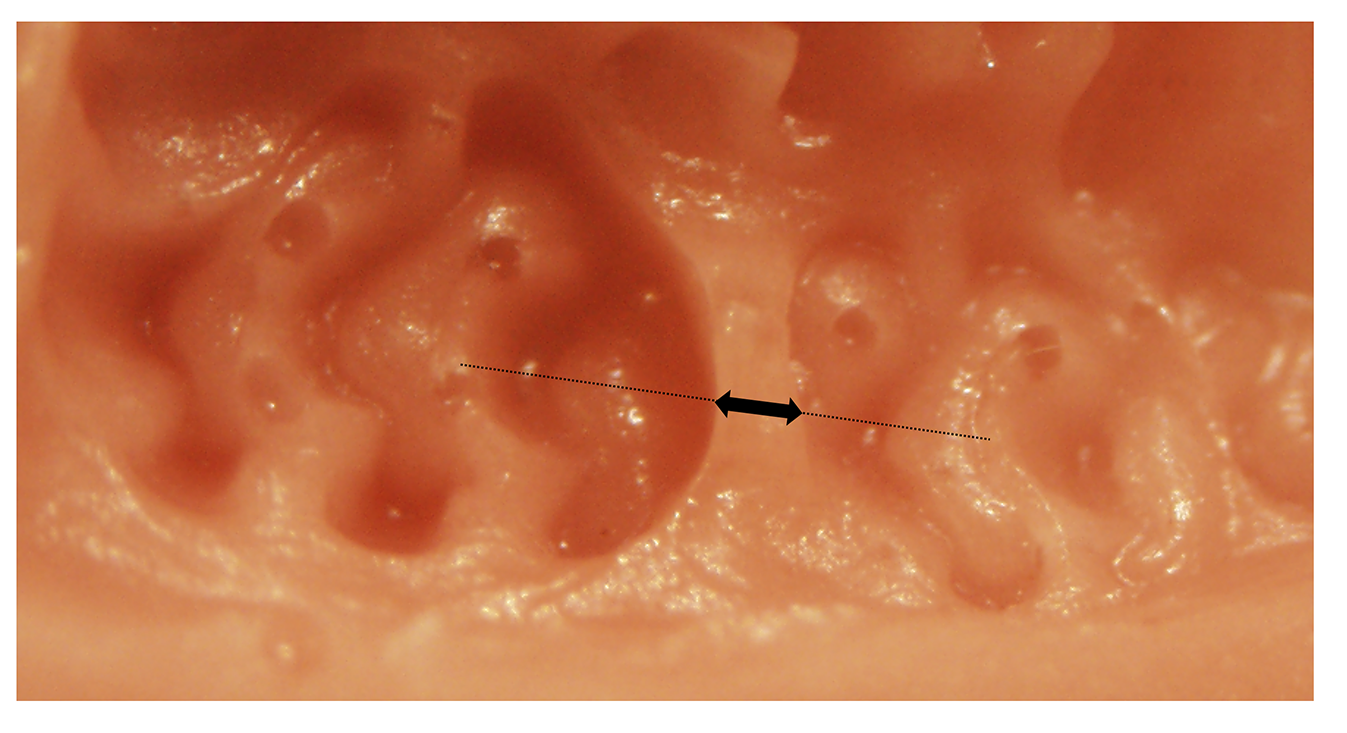

Supplement: S4 Fig — Image of the silicone impression (viewed under a stereoscopic microscope) taken after tooth movement for measurement of tooth movement. The amount of tooth movement was measured between the distal marginal ridge of the first molar to the mesial marginal ridge of the second molar at the level connecting the central fossae of the first and second molars (black double arrow). (TIF) [file pone.0223989.s004.tif]

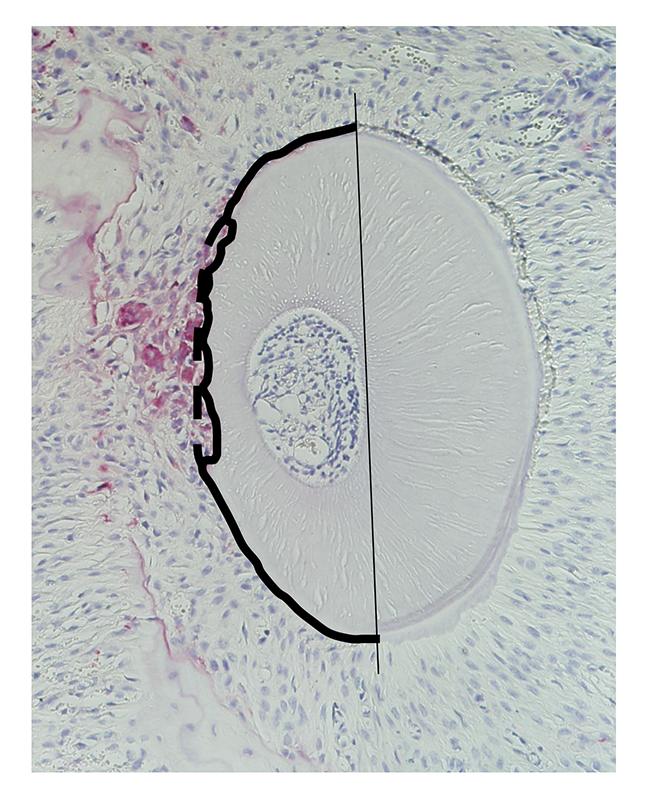

Supplement: S5 Fig — The image shows the evaluation of root surface resorption on transverse histological sections. The solid line represents the pressure side of the root surface and the interrupted line is the resorption surface. The root resorption surface was quantified by the percentage of the interrupted line/solid line. (TIF) [file pone.0223989.s005.tif]
